# Chimeric antigen receptor dendritic cells targeted delivery of a single tumoricidal factor for cancer immunotherapy

**DOI:** 10.1007/s00262-024-03788-1

**Published:** 2024-08-06

**Authors:** Rong Duan, Philip Milton, Chutamath Sittplangkoon, Xin Liu, Zhining Sui, Brendan F. Boyce, Zhenqiang Yao

**Affiliations:** 1 Department of Pathology and Laboratory Medicine, University of Rochester Medical Center, 601 Elmwood Ave, Rochester, NY 14642 USA; 2 School of Engineering, University of Rochester, Rochester, NY 14627 USA; 3 Department of Biostatistics and Computational Biology, University of Rochester Medical Center, 601 Elmwood Ave, Rochester, NY 14642 USA; 4 Present Address: Department of Orthopedics, Tianjin Hospital, Tianjin, 30021 People’s Republic of China

**Keywords:** Breast cancer, Dendritic cell (DC), Tumor necrosis factor-*α* (TNF), Chimeric antigen receptor (CAR) cell therapy, Mucin1, Inhibitor of apoptosis proteins (IAPs), IAP antagonist, Patient-derived xenograft (PDX) model

## Abstract

**Background:**

Chimeric antigen receptor (CAR)-T cells have been used to treat blood cancers by producing a wide variety of cytokines. However, they are not effective in treating solid cancers and can cause severe side-effects, including cytokine release syndrome. TNF*α* is a tumoricidal cytokine, but it markedly increases the protein levels of cIAP1 and cIAP2, the members of inhibitor of apoptosis protein (IAP) family of E3 ubiquitin ligase that limits caspase-induced apoptosis. Degradation of IAP proteins by an IAP antagonist does not effectively kill cancer cells but enables TNF*α* to strongly induce cancer cell apoptosis. It would be a promising approach to treat cancers by targeted delivery of TNF*α* through an inactive adoptive cell in combination with an IAP antagonist.

**Methods:**

Human dendritic cells (DCs) were engineered to express a single tumoricidal factor, TNF*α*, and a membrane-anchored Mucin1 antibody scFv, named Mucin 1 directed DCs expressing TNF*α* (M-DCs^TNF^). The efficacy of M-DCs^TNF^ in recognizing and treating breast cancer was tested in vitro and in vivo.

**Results:**

Mucin1 was highly expressed on the surface of a wide range of human breast cancer cell lines. M-DCs^TNF^ directly associated with MDA-MB-231 cells in the bone of NSG mice. M-DCs^TNF^ plus an IAP antagonist, SM-164, but neither alone, markedly induce MDA-MB-231 breast cancer cell apoptosis, which was blocked by TNF antibody. Importantly, M-DCs^TNF^ combined with SM-164, but not SM-164 alone, inhibited the growth of patient-derived breast cancer in NSG mice.

**Conclusion:**

An adoptive cell targeting delivery of TNF*α* combined with an IAP antagonist is a novel effective approach to treat breast cancer and could be expanded to treat other solid cancers. Unlike CAR-T cell, this novel adoptive cell is not activated to produce a wide variety of cytokines, except for additional overexpressed TNF, and thus could avoid the severe side effects such as cytokine release syndrome.

## Introduction

Adoptive cell therapies (ACTs), such as genetically modified T cells expressing novel T cell receptors or chimeric antigen receptors (CARs), have revolutionized cancer treatment. The CARs recognize and bind to specific antigens on the surface of cancer cells. Six CAR-T cell therapies have been approved by FDA to treat blood cancers, including lymphomas, some forms of leukemia, and multiple myeloma since 2017 [1]. But they are not effective in treating solid cancers.

The efficacy of the ACT in treating cancers depends on the engineered cell production of a wide variety of cytokines, including interleukin-IL-2 (IL-2), IL-4, IL-6, TGF-*β*, interferon-*γ*, tumor necrosis factor-*α* (TNF), IL-8, and IL-10, IL-15, IL-18, IL-21, etc. [2]. One strategy to augment the antitumor efficacy of CAR-T cells is to design one or more intracellular signaling domains from various costimulatory protein receptors, such as CD28 and 41BB, incorporated in the cytoplasmic tail of the CAR, as did in second and third generation of CARs, to promote and enhance TCR signaling [3]. However, like all cancer treatments, CAR-T cell therapies can cause severe side effects, including a mass die-off of antibody-producing B cells and infections, one of the most frequent and serious which is cytokine release syndrome (CRS) due to the massive production of cytokines [4]. The fourth-generation CAR-T is trying to modulate the tumor environment through an inducible release of transgenic immune modifier, such as IL-12, to enhance their antitumor efficacy [5].

The fifth- or next-generation CAR constructs have been designed to enhance their ability to kill cancers while reducing toxic, for example, (1) ON-switch CARs with split receptors to control T cell function by a small molecule; (2) the split, universal, and programmable (SUPRA) CAR system to finely tune T cell activation strength and logically respond to multiple antigens; and (3) combinatorial antigen-sensing circuits for precise therapeutic discrimination through a second tumor antigen [6, 7]. Like CAR-T cells, CAR-NK cells have been developed by engineering the NK cells with extracellular, transmembrane and intracellular signaling domains [7]. CAR-macrophages have also been developed to kill cancers by producing pro-inflammatory cytokines and chemokines, polarizing M2 to M1 macrophages and boosting T cell function [8]. It is promising to develop a new class of adoptive cell that produces a unique single tumoricidal factor to treat cancers while avoiding the severe side effects including CRS.

TNF*α* was originally identified as a tumoricidal cytokine [9]. But no cancer patients responded to i.v. infusion of recombinant TNF*α* in phase II clinical trials [10, 11]. As a pro-inflammatory cytokine produced by both tumor and host cells, TNF*α* is implicated in inflammation and subsequent tumor development and progression [12]. Now it is clear that TNF*α* determines the fate of cancers based on cancer expression of inhibitor of apoptosis proteins (IAPs) family of E3 ubiquitin ligases, including cIAP1 (BIRC2), cIAP2 (BIRC3), X-IAP (BIRC4), etc. IAP proteins, which are frequently overexpressed in human tumors and promote cancer cell survival [13], induce proteasomal degradation of caspases to block apoptosis [14, 15]. TNF*α* alone does not effectively kill cancer cells because it markedly increases protein levels of cIAP1 and cIAP2 by cancer cells [16, 17].

Endogenous Second Mitochondria-Derived Activators of Caspase (SMAC) can degrade IAPs, resulting in caspase activation and triggering apoptosis [18]. Many synthesized SMAC mimetics, also called IAP antagonists, have been developed to degrade IAPs for cancer therapy. Some of them, such as GDC-0917, LCL161, AT-406, HGS1029, and TL32711, have been studied in phase I or II clinical trials, but most patients did not benefit from them [19–24]. An IAP antagonist alone, such as SM-164, AT-406, or BV6, which effectively degrades IAPs, does not effectively induce cancer apoptosis, but enables TNF*α* to strongly induce cancer cell apoptosis [16]. Thus, targeted delivery of TNF through an inactive adoptive cell in combination with an IAP antagonist could be a novel approach to treat cancers while obviating the severe side effects from both classical ACT therapy and the systemic administration of TNF*α*.

## Materials and methods

### Animals

Female athymic nude mice and immunocompromised NOD/SCID gamma (NSG) mice (NOD.Cg-Prkdc^scid^ Il2rg^tm1Wjl^/SzJ) originally were purchased from Jackson Laboratory, and NSG mice were bred in house. All experimental protocols were approved by the University of Rochester Committee for Animal Resources. All methods were carried out in accordance with the American Veterinary Medical Association (AVMA) guidelines and regulations.

### Construction of the plasmids

A cDNA of Muc1 monoclonal antibody, scFv [25, 26], linked to human CD66b transmembrane/cytoplasmic domain through a hinge, called chimeric CD66b-scFv, was synthesized (Fig. 2B) and was inserted into a pLV lentiviral vector expressing mCherry, to construct a pLV^CD66b−scFv−mCherry^ plasmid. The expressions of CD66b-scFv and mCherry are driven by two different promoters. Similarly, a synthesized cDNA of hTNF*α* was inserted into pLV vector to construct a pLV^hTNF^ plasmid. All these plasmids were generated in DH5*α* cells. 293TN cells (ATCC CRL-3216) were infected with the lentiviral plasmid with a packaging reagent kit (OriGene TR30037) to generate lentiviral particle supernatant.

### Cell transduction

Fifteen milliliters of peripheral blood from adult healthy donor was incubated with NH4Cl solution to lyse red blood cells and to generate peripheral blood mononuclear cells (PBMCs). The protocol using human materials was approved by the University of Rochester Research Subjects Review Board. The PBMCs were cultured with 5 ng/ml of M-CSF (R&D System, Cat# 216-MC-010) and GM-CSF (R&D System, Cat# 215-GM-010) for 5 days to generate DCs, which were incubated with 1:1 combination of CD66b-scFv and hTNF*α* lentivirus, or single CD66b-scFv lentivirus for 24 h. The culture medium with lentivirus was removed, and the cells were maintained with monocyte culture medium with M-CSF and GM-CSF for 4 additional days. Culture medium was collected to measure the hTNF*α* concentration and to test its efficacy to kill cancer cells in combination with an IAP antagonist. Adoptive DCs attached to the dishes were harvested through trypsin digestion for injection and cell phenotype analysis.

### M-DCs^TNF^ recognition of breast cancer cells

DCs derived from PBMCs through M-CSF and GM-CSF treatment were infected with pLV^CD66b−scFv−cherry^ and control pLV^mCherry^ lentivirus to generate DCs^CD66b−scFv−cherry^ and DCs^mCherry^, respectively. The expression of the tagged mCherry, tested by flow cytometry, was used to evaluate to the % of DCs expressing chimeric CD66-Muc1-scFv. The viral titer (transducing units per mL (TU/mL)) was calculated by the formula: the number of mCherry^+^ cells/total volume in the well (mL) × dilution factor. Multiplicity of infection (MOI) was calculated using the following formula: [Virus titer (TU/mL) × lentiviral volume used for transduction (mL) × dilution factor]/(# of cells at transduction). GFP^+^ MDA-MB-231 cells were injected into the tibial marrow cavity of NSG mice, and DCs^CD66b−scFv−cherry^ and DCs^mCherry^ were injected into the same marrow cavity 5 days later. After 3 days, the mice were euthanized, and their bones were processed for frozen sectioning to test for interactions between cancer cells and DCs expressing chimeric CD66b-scFv in comparison with those expressing mCherry using fluorescent microscopy.

### ELISA testing of TNF*α*

Levels of TNF*α* in culture medium were tested by ELISA, according to the manufacturer’s instructions (Invitrogen, #KHC3011).

### Flow cytometry

To test cell surface markers, 1 × 10^6^ cells freshly isolated cells from blood (tissue) or cultured cells, digested using 0.25% trypsin on the culture dishes, from each sample were stained with fluorescence-conjugated antibodies in FACS buffer (2% FBS in PBS) at 4 °C for 30 min. To test the expression of TNF, the cells were treated with 2 μl Golgi stop (BD Biosciences Cat# 554724) per dish in the last 12 h. The cells in FACS buffer were then immediately subjected to analysis using a flow cytometer (FACS LSR II; BD Biosciences). FlowJo software was used for data analysis.

### Apoptosis assay

MDA-MB-231 cells were treated with different doses of SM-164 ± conditional medium, TNF antibody (BioX Cell, #BE0058) or 1 ng/ml TNF*α* (R&D Systems, Cat# 210-GMP) for 24 h. The cells were stained with anti-Annexin V Ab (eBioscience, REF# 11–8005, or BioLegend Cat# 640941) and propidium iodide (PI, BioLegend Cat# 421301) and subjected to FACS to analyze Annexin V^+^PI^±^ apoptotic cells.

### Xenograft and therapeutic evaluation of human breast cancer in mice

To investigate the effects of IAP antagonists and related agents on breast cancer-induced osteolysis, 1 × 10^5^ MDA-MB-231 cells bearing luciferase [16] in 5 *μ*l PBS was directly injected into tibial bone marrow cavities through the tibial plateau of NSG mice and the tumor growth was monitored by measuring bioluminescence imaging (BLI) signal in the legs. To determine whether depletion of macrophage influences the effect of IAP antagonist in treating BC growth in bone, 0.25 ml Clodronate Liposomes (Tribioscience, #F70101C-A) was I.P. injected to an adult NSG mouse. At the end of experiments, mice were euthanized, legs were harvested to analyze cancer-induced osteolysis (trabecular bone volume, BV/TV) by micro-CT, as we reported recently [27, 28], followed by histological analysis of tumor burden, and internal organs were harvested to examine for tumors histologically [16, 17]. To investigate the effect of M-DC^TNF^ and IAP antagonists on TNBC, 50 *μ*l of minced tumor tissue from a NSG mouse bearing the established PDX model of human TNBC (Jax Lab #TM00090) were injected subcutaneously into the left flank of NSG mice. Tumor size was measured by a digital caliper and tumor weight was quantified by the end of the experiment. Using human breast cancer tissue to develop PDX model was approved by University of Rochester Research Subjects Review Board.

### Statistics

Descriptive statistics are presented by means and standard deviations for continuous variables. When data distributions were skewed, median and interquartile ranges were used. In addition, frequencies were presented for categorical variables. Comparisons between two groups were analyzed using Student’s two-tailed unpaired *t* test and among three or more groups using one-way analysis of variance followed by Dunnett’s post-hoc multiple comparisons. When data distributions were not normal, Kruskal–Wallis testing was used to compare medians. Nonparametric statistical analyses were used for comparisons of frequency of bone and lung metastasis in mice. All analyses were performed at a two-tailed 0.05 significance level. The power analysis was performed based on the ANOVA test. According to our previous data [16], the means of tumor volume in the long bone in vehicle, SM-164, standard chemotherapy and BV6 were 6.55, 0.28, 1.19 and 4.45 (mm^2^) with 3.78, 0.71, 1.58 and 2.37 (mm^2^) SD, respectively. Seven subjects (initially 8 to ensure enough subjects at the end) will be needed in each group to achieve at least 85% power at 0.05 two-tailed significance level.

## Results

**Fig.1:**
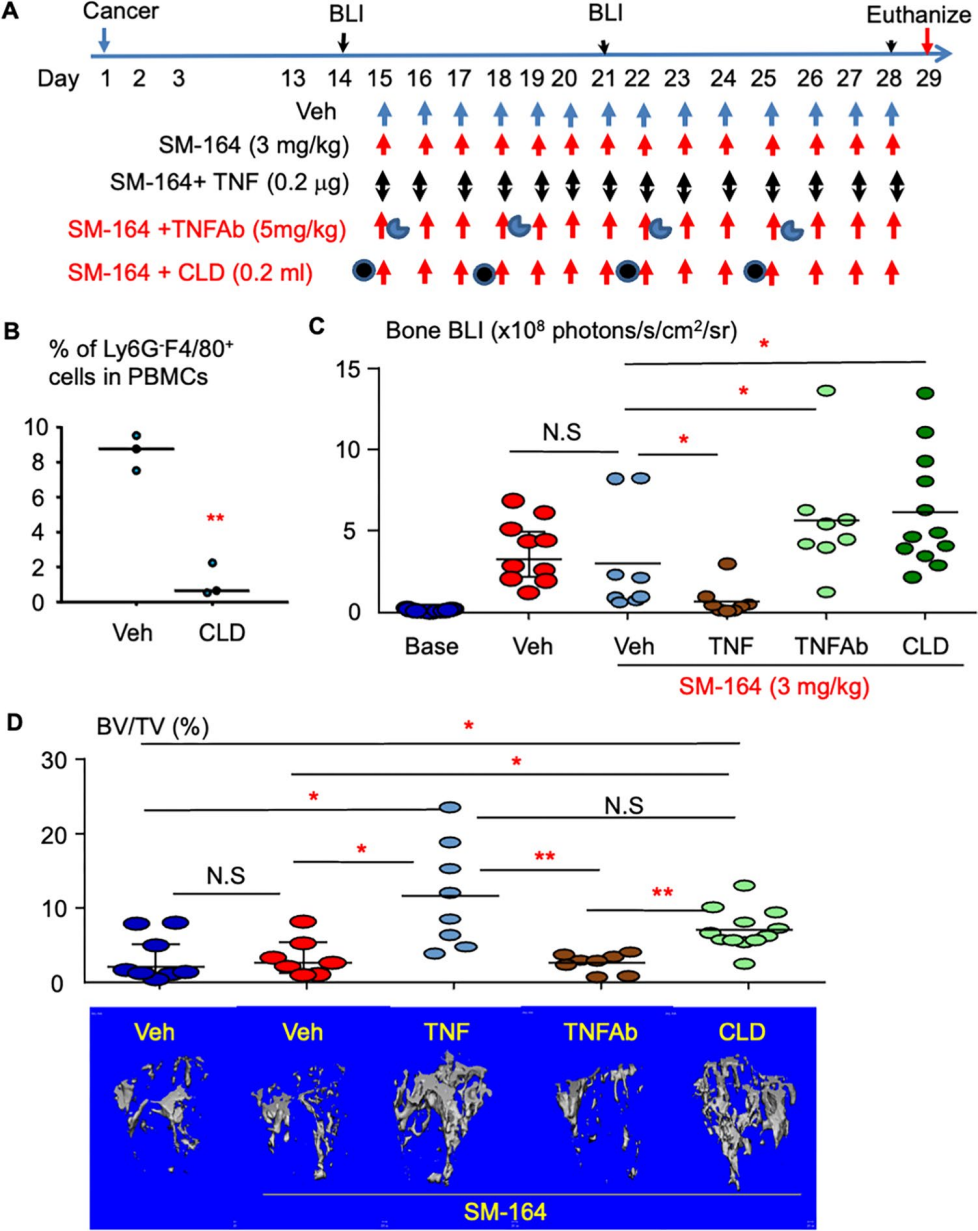
Endogenous TNF*α* is important for and exogenous TNF*α* enhances the effect of an IAP antagonist to reduce the growth of breast cancer in bone. **A** Scheme showing schedule of macrophage depletion and TNF blockade during SM-164 treatment of established breast cancer metastases in bone; 4–5 mice (8–10 samples) per group. **B** Efficacy of CLD to deplete circulating macrophages. The data were from three mice per group. **C** BLI signal intensity of breast tumor in tibiae, assessed two weeks after treatment. **D** Trabecular bone mass in tibiae by micro-CT showing the effect of TNF blockade and macrophage depletion on SM-164 treatment of breast cancer-induced osteolysis. **p < 0.01, unpaired T test in (**B**); *p < 0.05, **p < 0.01, ANOVA^+^/Dunnett test, based on logarithm-transformed intensity in (**C**) and (**D**)

Myeloid cells, including monocytes, macrophage and DCs, are the main cellular sources of TNF*α*, and macrophages are the most abundant support cell in the tumor microenvironment [29]. We first tested if myeloid cell production of TNF*α* is critical for, and if systemic administration of TNF*α* can enhance the effect of, an IAP antagonist in treating advanced metastasis from breast cancer, the second leading cause of cancer death in women. MDA-MB-231 cells were directly injected into the bone marrow cavity of right tibiae of NSG mice. After 2 weeks when the tumors were established in bone, assessed by bioluminescence imaging (BLI) signals, the mice were randomly divided into five treatment groups: Vehicle; SM-164 alone; or SM-164 plus either TNF*α*, TNF antibody (Ab), or clodronate liposomes (CLD), as illustrated in Fig. 1A. CLD effectively depleted Ly6G^−^F4/80^+^ macrophages (Fig. 1B). Two weeks after treatment, BLI signals were tested to monitor the tumor growth. The results indicated that SM-164 plus TNF*α*, but not SM-164 alone, significantly reduced tumor BLI signal levels in the bone (Fig. 1C). Of note, SM-164 plus TNF Ab or CLD slightly but significantly increased tumor BLI levels in bone compared to SM-164 alone or SM-164 plus TNF (Fig. 1C), suggesting that blocking TNF signaling or depletion of macrophages attenuated the effect of SM-164 in inhibiting the growth of breast cancer in bone although SM-164 alone did not significantly reduce the growth of this advanced stage breast cancer.

Three weeks after treatment, the mice were euthanized and the bones were harvested to analyze the bone mass by micro-CT. SM-164 alone did not increase trabecular bone volume in the bones with cancer, as assessed by micro-CT scanning (Fig. 1D). In contrast, SM164 plus TNF*α* markedly increased trabecular bone volume in the bones with cancer, suggesting that their combined therapy protected these bones from cancer-induced osteolysis (Fig. 1D). Addition of TNF Ab appeared to not change the trabecular bone volume compared to SM-164 alone. The tibial trabecular bone mass in mice treated with SM-164 plus CLD was also significantly higher than those in mice treated with vehicle or SM-164 alone (Fig. 1D), although it increased tumor growth (Fig. 1B), because CLD depletes macrophages (Fig. 1B) and promotes osteoclast apoptosis [30] to inhibit bone resorption. These findings suggest that administration of exogenous TNF plus SM-164, but not SM-164 alone, effectively inhibits the growth of metastatic breast cancer in bone and protects from cancer-induced osteolysis.

**Fig.2:**
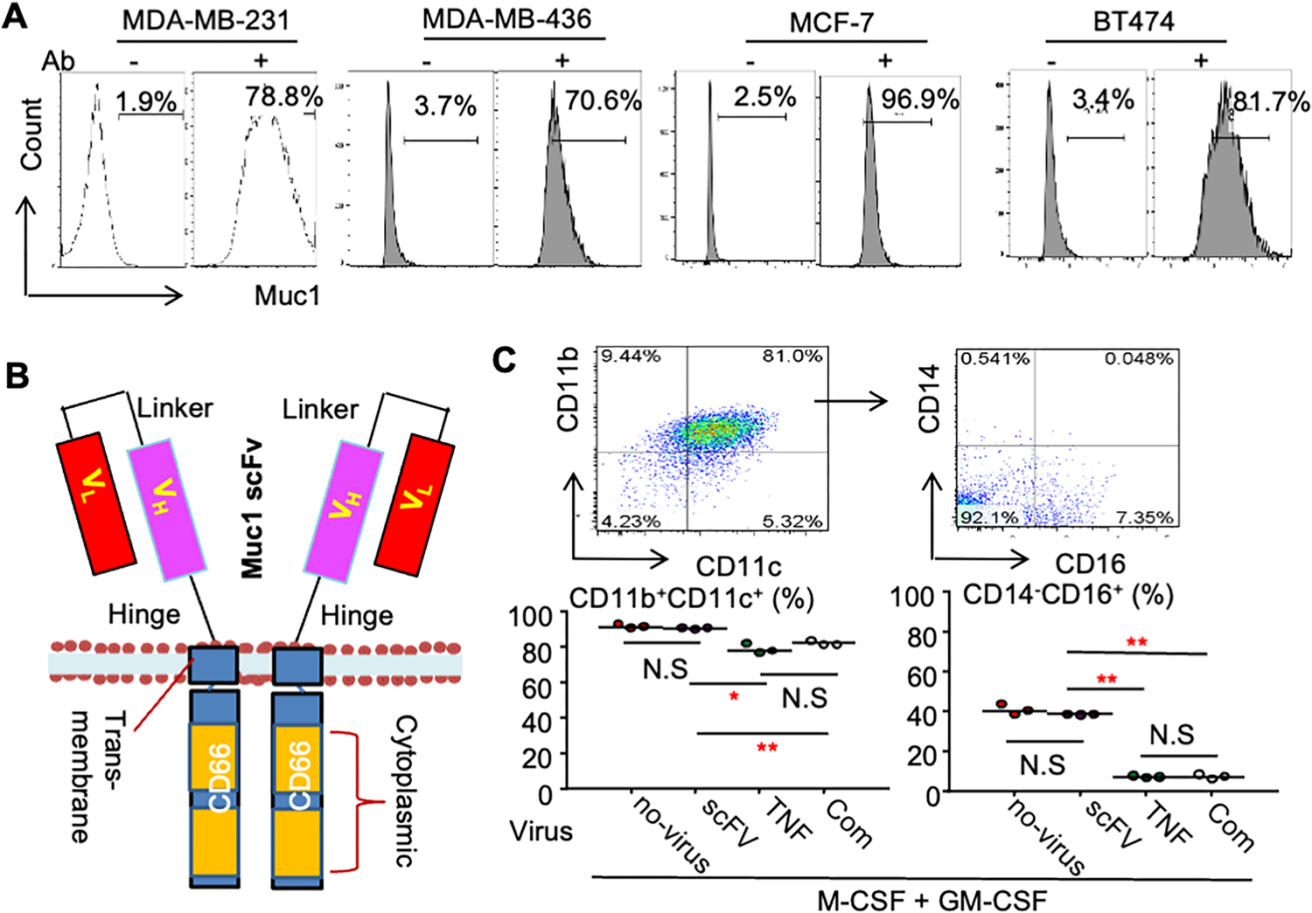
Design of Mucin1-directed DCs. **A** Flow cytometry showing a variety of breast cancer cell lines expressing Mucin1. **B** Design of chimeric CD66b-Muc1 monoclonal Ab, scFv, by linking Muc1 Ab scFv to the transmembrane/intracellular domain of CD66b. **C** Phenotype analysis of M-DCs^TNF^ by flow cytometry. 3 × 10^6^ of human PBMCs were cultured with 3 ng/ml of GM-CSF and M-CSF in a 60-mm dish for 6 days to generate DCs (3 × 10^4^), which were then infected with 0.5 ml of indicated lentiviral supernatant for 2 days. The indicated cell surface markers of the DCs were analyzed by flow cytometry. The data were from three repeats in (**C)**. *p < 0.05, **p < 0.01, ANOVA^+^/Dunnett test

TNF*α* can cause serious side effects in humans, including systemic shock and inflammatory reactions [10, 11, 31]. Therefore, targeting tumors to deliver TNF*α* locally, combined with an IAP antagonist, would be an attractive approach to eliminate cancers strongly and specifically with fewer side-effects. The first thing to develop a targeted therapy is to find a molecule that is highly expressed on the cancers. One of the molecule candidates to target cancers is Mucin 1 (Muc1), a glycoprotein expressed on the surface of epithelial cells, and its overexpression is often associated with tumorigenesis and metastases, including breast [32–34], pancreatic [35] and lung cancers [36]. We confirmed that a broad range of breast cancer cell lines, including triple-negative breast cancer (TNBC) cell lines MDA-MB-231 and MDA-MB-436 cells, estrogen positive (ER^+^) MCF7 cells and HER2 positive (Her2^+^) BT474 cells, highly expressed Muc1 protein on their surface (Fig. 2A), suggesting that Muc1 is a good target, at least, for a broad range of breast cancers.

Macrophage or DC was selected as a tool to targeted deliver TNF to cancers because they are abundant support cells in the tumor microenvironment [29] and are terminal differentiated cells and their life span can be weeks to months [37, 38]. CD66b, also named carcinoembryonic antigen-related cell adhesion molecule 8 (CEACAM8), was chosen to construct a CAR because it is expressed by monocytes and granulocytes to regulate their adhesion and activation [39, 40]. We constructed a chimeric CD66b-Muc1 scFv cDNA by replacing the extracellular domain of CD66b with a cDNA of the Muc1 monoclonal Ab, scFv (Fig. 2B). Peripheral blood mononuclear cells (PBMCs) from a healthy adult subject were cultured with M-CSF and GM-CSF for 5 days, followed by co-infection with a combination of pLV^CD66b−scFv−cherry^ and pLV^hTNF^ lentiviruses to generate adoptive DCs expressing scFv on their surface and secreting TNF*α*, which we called Muc1-directed DCs producing TNF*α* (M-DCs^TNF^). M-DCs^TNF^ highly express both CD11b and CD11c, but not the monocyte marker, CD14 (Fig. 2C). In addition, they have reduced expression of the monocyte cell marker, CD16, compared to DCs induced by GM-CSF/M-CSF (Fig. 2C). Reduced CD16 expression on M-DCs^TNF^ was attributed to the effects of overexpression of TNF*α* because overexpression of CD66b-scFv in these cells did not change their phenotype (Fig. 2C).

**Fig.3:**
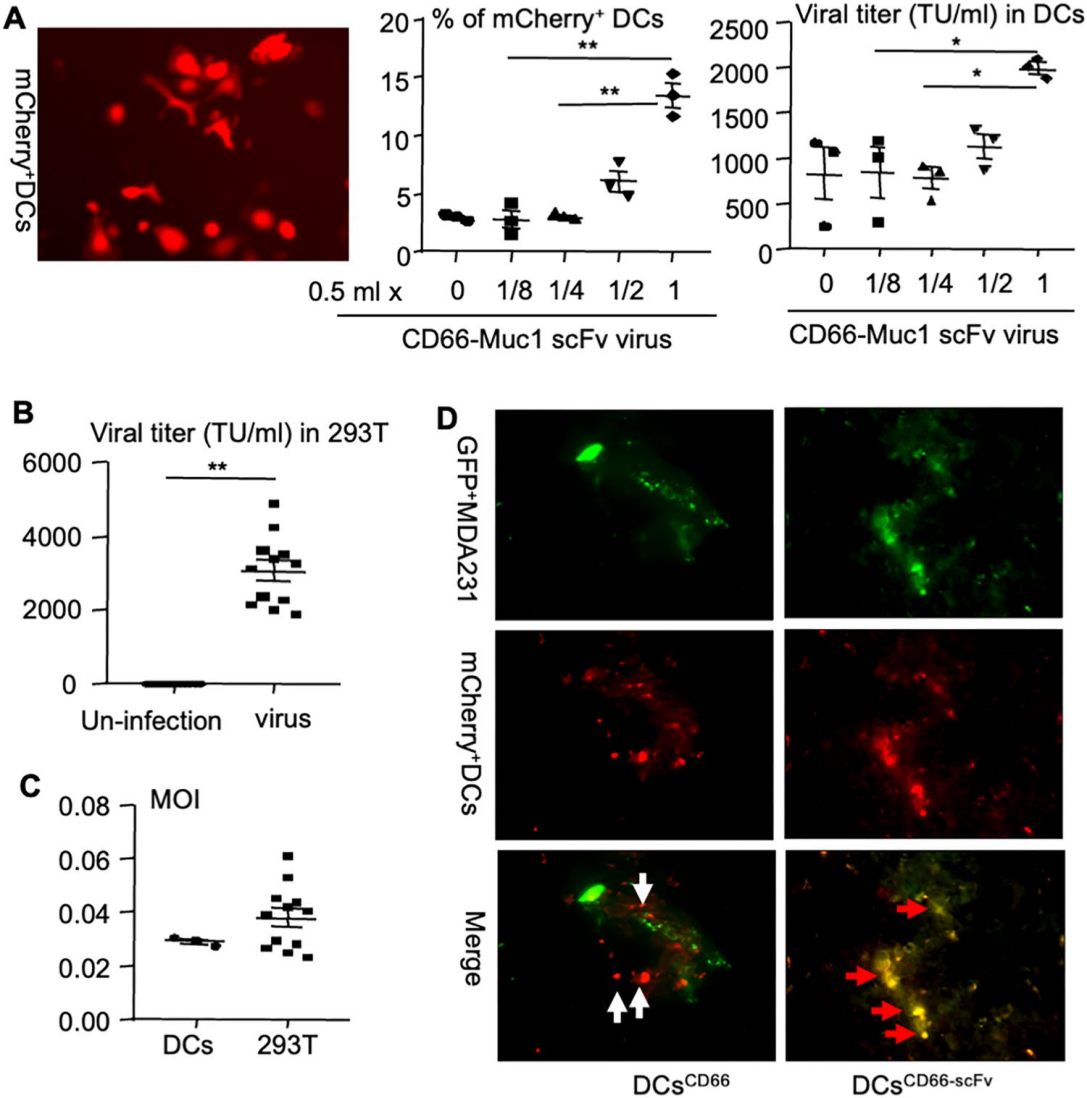
Validation of Mucin1-directed DCs targeting breast cancer. **A** The chimeric CD66-Muc1 scFv-mCherry lentiviral supernatant was added to the 3 × 10^4^ of cultured DCs, generated as in Fig. 2C, in a 60-mm dish with total of 2 ml culture medium for 1 day and continued to culture 2 more days after the virus was removed. mCherry^+^ cells were shown by microscopy (left panel), the % of mCherry^+^ cells were analyzed by flow cytometry (middle panel), and the viral titer was calculated (right panel). **B** HEK293T cells (6 × 10^4^ cells/well in 96-well plate) were used to test the titer of chimeric CD66-Muc1 scFv-mCherry lentivirus by adding 30 μl of fivefold serial diluted viral supernatant for 5 h followed by culturing 3 days with alpha-MEM containing 10% FBS at 37 °C incubator. **C** Multiplicity of infection (MOI) for DCs and HEK293T cells, tested as in above **A** and **B**, was calculated. **D** 5 × 10^4^ of GFP^+^ MDA-MB-231 cells and 2 × 10^5^ of DCs expressing mCherry-tagged chimeric CD66-Muc1 scFv or control DCs expressing mCherry without Muc1 scFv were injected into the tibial marrow cavity of NSG mice on day 1 and 6, respectively. Mice were euthanized on day 9 to test interactions of cancer cells with DCs in frozen sections of tibiae using fluorescent microscopy. All titrations were carried out at least three replicates in (**A**) and (**B**). *p < 0.05, **p < 0.01, ANOVA^+^/Dunnett test

To test whether M-DCs^TNF^ can recognize breast cancer cells, we first tested efficacy of M-DCs^TNF^ expressing the chimeric CD66b-Muc1 scFv tagged with a mCherry. The results indicated that 0.5 ml (1/4 of total culture medium) of the viral particles caused about 14% of the DCs to express mCherry/ CD66b-Muc1 scFv (Fig. 3A middle panel) and viral titer was about 2000 TU/ml (Fig. 3A right panel). This viral volume was the lowest effective volume to infect the DCs to express chimeric CD66b-Muc1 scFv/mCherry since 0.25 ml or lower viral volume did not cause significant expression of mCherry (Fig. 3A middle and right panel). Similarly, the viral titer of chimeric CD66-Muc1 scFv-mCherry lentivirus, tested using HEK293T cells, ranged from 2000 to 5000 (median 3,188) TU/ml (Fig. 3B). The MOI of the virus, tested using DCs, was about 0.03, while it ranged from 0.03 to 0.06 (median 0.04), tested using 293 T cells (Fig. 3C). To test whether M-DCs^TNF^ can recognize breast cancer cells in vivo, GFP^+^ MDA-MB-231 cells and DCs expressing CD66b-scFv-cherry or CD66b-cherry were injected into the tibial marrow cavity of athymic nude mice on day 1 and 6, respectively. Mice were euthanized on day 9 and their bones were processed for frozen sectioning to test for interactions between DCs expressing chimeric CD66b-scFv and breast cancer cells using fluorescent microscopy. Compared to DCs^CD66b−cherry^, which did not associate with MDA-MB-231 cells, most DCs^CD66−scFv−cherry^ bound to GFP^+^ MDA-MB-231 cells and became yellow (Fig. 3D), confirming that DCs^CD66−scFv−cherry^ specifically recognized and attached to MDA-MB-231 cells in vivo.

**Fig.4:**
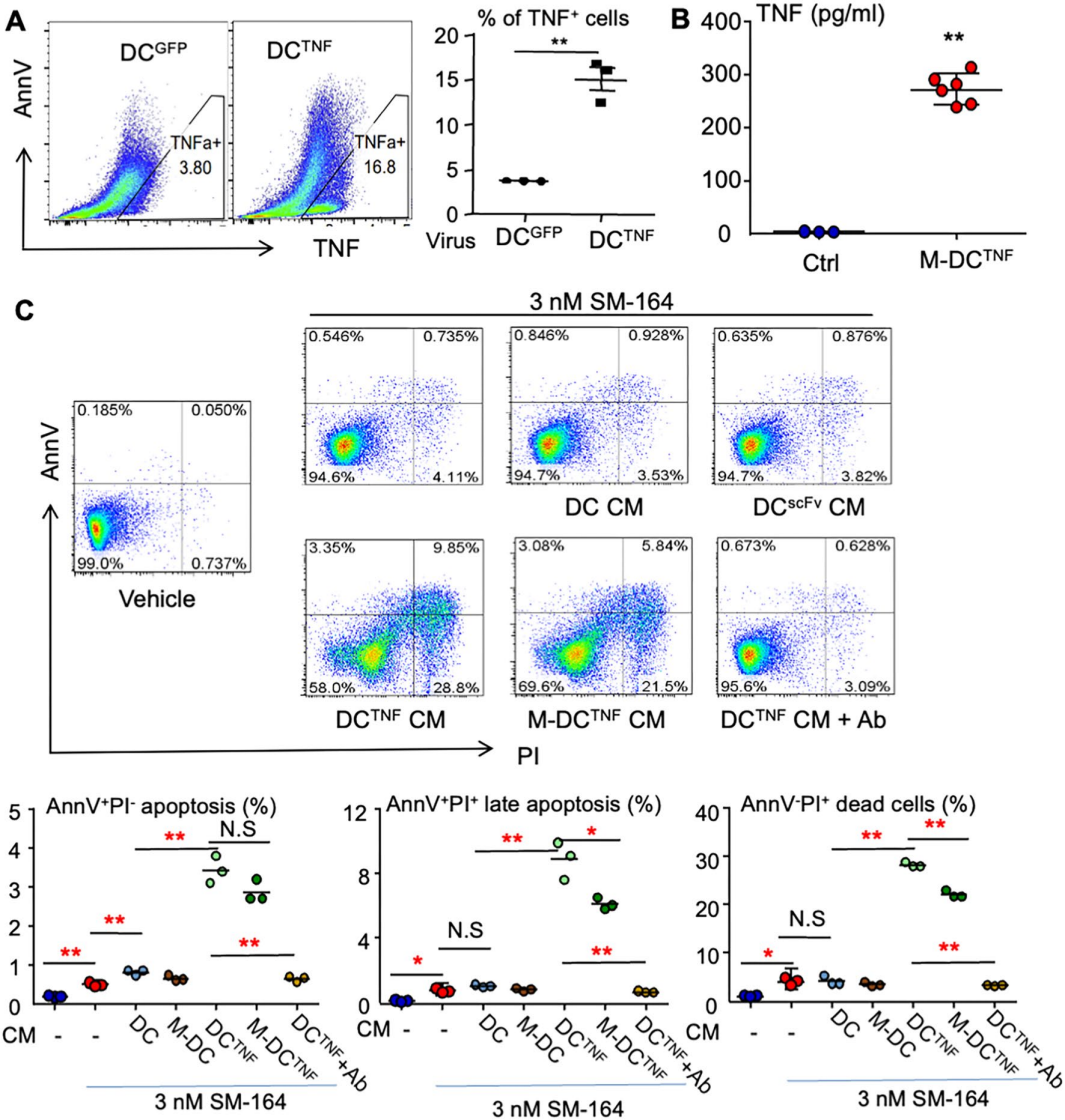
M-DCs^TNF^ in combination with SM-164 kill cancer cells by secreting hTNF in vitro. DCs derived from human PBMCs by M-CSF and GM-CSF were infected with chimeric CD66b-Muc1 monoclonal Ab scFv and hTNF lentivirus to generate M-DCs^TNF^, as in Fig. 2 (**C**) & 3 (**A**). **A** The cells in a 60-mm dish were treated with 2 μl Golgi stop in last 12 h. TNF^+^ cells were analyzed by flow cytometry. **B** Condition medium (CM) from cultured M-DCs^TNF^ was used to test hTNF levels by ELISA. Control (ctrl)=Breast cancer cell culture medium. **C** Flow cytometry analysis showing CM from cultured M-DCs^TNF^ effectively kills MDA-MB-231 cells in combination with SM-164. The data were from three repeats. **p < 0.01, unpaired T test in (**A**); *p < 0.05, **p < 0.01, ANOVA^+^/Dunnett test in (**C**). M-DC^TNF^=DC^scFv−TNF^, Ab=human TNF antibody

We then tested whether M-DCs^TNF^ produce TNF*α* and kill breast cancer cells in combination with an IAP antagonist. We first tested the % of M-DCs^TNF^ expressing TNF by flow cytometry. The results showed that 0.5 ml of the viral supernatant infection yielded 15% of the DCs expressing TNF (Fig. 4A), with similar infection efficacy with chimeric CD66b-Muc1 scFv (Fig. 3A). We then used ELISA to test human TNF*α* levels in the conditioned mediums (CM) from cultured M-DCs^TNF^ or control cells. The results showed that the CM indeed had high levels of TNF*α* (Fig. 4B). As expected, 3 nM of SM-164 alone slightly but significantly increased the % of AnnV^+^PI^−^ early and AnnV^+^PI^+^ late apoptosis and AnnV^−^PI^+^ dead cells after overnight treatment (Fig. 4C). Addition of CM from cultured normal DCs slightly increased the % of SM-164-induced early- but not late-stage apoptotic and dead cells (Fig. 4C). Importantly, addition of CM from cultured M-DCs^TNF^ effectively killed MDA-MB-231 cells when 3 nM of SM-164 was added, and total apoptotic (AnnV^+^PI^−^ early and AnnV^+^PI^+^ late apoptosis) and dead (AnnV^−^PI^+^) cells was about 40% after overnight treatment (Fig. 4C). CM from co-infection with both vectors expressing chimeric CD11b-scFv and TNF slightly reduced the % of AnnV^+^PI^+^ late apoptotic and AnnV^−^PI^+^ dead cells caused by SM-164 (Fig. 4C), suggesting that co-infection with both vectors may reduce the infection efficacy for each alone. Interestingly, the cancer cell apoptosis and death caused by M-DCs^TNF^ CM plus SM-164 were completely blocked by anti-TNF antibody (Fig. 4C). This confirms that M-DCs^TNF^ CM kills the cancer cells through TNF in the presence of an IAP antagonist. In contrast, CM from DCs infected with chimeric CD66b-scFv lentivirus alone (M-DCs) did not cause cancer cell death when SM-164 was added.

**Fig. 5:**
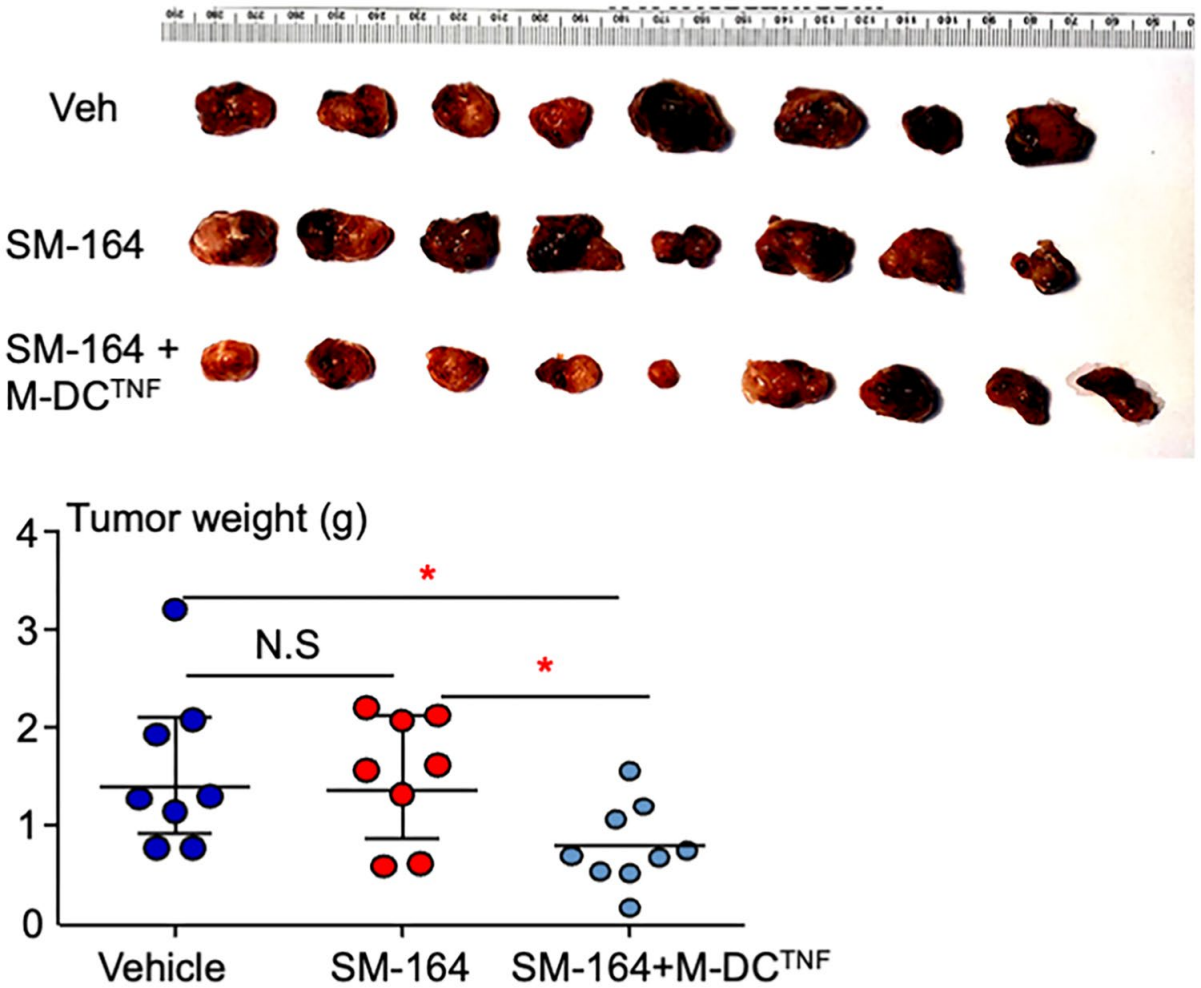
Effects of M-DCs^TNF^ combined with SM-164 in treatment of TNBC PDX model. 50 μl of minced tumor tissue from PDX model of breast cancer (Jax Lab #TM00090) were injected subcutaneously in the left flank of NSG mice. After two weeks when the tumors were ~ 0.5 cm, mice were injected with 1 × 10^5^ M-DC^TNF^ or PBS followed by treatment with vehicle or 3 mg/kg SM-164, twice a day for 10 days. Eight mice per group. Mice were euthanized, and the tumors were weighed. *p < 0.05, ANOVA + /Dunnett test

We used a patient-derived xenograft (PDX) model to test the therapeutic response of established breast cancer to M-DCs^TNF^ + SM-164 in NSG mice. We harvested tumors from a NSG mouse transplanted with TNBC tumor from a patient (Jax Lab #TM00090), cut the tumors into small pieces (1–2 mm) and injected 50 μl of them subcutaneously into the left flank of NSG mice (8–9 mice per group). After 2 weeks, when the tumors were about 0.5 cm in diameter, the mice were randomly divided three groups and 2 × 10^5^ M-DCs^TNF^ were subcutaneously injected around the tumors one time in one group of mice. After 2 days, the mice were treated with vehicle or 3 mg/kg of SM-164 twice a day. Two weeks later, the mice were euthanized, and tumors were weighed. SM-164 alone had no effect on tumor size or weight compared to the vehicle (Fig. 5). This is consistent with the facts that (1) an IAP antagonist alone, which effectively degrades IAP proteins, does not kill cancer [16], and (2) the endogenous TNF levels are too low in the mice and do not efficiently trigger cancer cell apoptosis when IAP proteins are degraded by an IAP antagonist (Fig. 1). In contrast, M-DCs^TNF^ plus SM-164 significantly reduced tumor size and weight compared to either vehicle or SM-164 alone (Fig. 5).

## Discussion

We have developed a novel adoptive dendritic cell, M-DC^TNF^, which expresses membrane-anchored Muc1 monoclonal Ab, scFv, to target a broad range of breast cancers and produces a single tumoricidal factor-TNF*α* locally to kill cancer cells in combination with the IAP antagonist, SM-164, which degrades IAP proteins [16]. We confirm that DC^CD66b−scFv−cherry^ cells express the chimeric CD66b-Muc1 Ab scFv on their surface (Fig. 3A). Importantly, injected DC^CD66b−scFv−cherry^ cells directly associate with GFP^+^ MDA-MB-231 cells in the bones of athymic nude mice (Fig. 3D), indicating that this engineered DC specifically recognizes breast cancer cells. We also confirm that M-DCs^TNF^ express TNF*α* (Fig. 4A) and culture medium from M-DCs^TNF^ contains high levels of hTNF*α* (Fig. 4B). Importantly, the culture medium from M-DCs^TNF^ rapidly induces apoptosis of MDA-MB-231 cells in the presence of SM-164 and the cell death was completely blocked by addition of TNF antibody (Fig. 4C). These confirm the concept that M-DCs^TNF^ target and kill breast cancer by producing TNF*α* when an IAP antagonist is present. Importantly, a one-time injection of M-DCs^TNF^ combined with SM-164, but not SM-164 alone, effectively inhibits the growth of TNBC in a PDX mouse model (Fig. 5).

An important feature of M-DCs^TNF^ is that they are not activated and do not cause a cytokine release syndrome. M-DCs^TNF^ alone do not kill cancer cells and function solely as a tool to recognize and attach to cancers and produce TNF*α* locally. SM-164, which degrades IAP proteins, enables M-DCs^TNF^ to kill cancer cells specifically and effectively. CD66b is expressed on myeloid cells and functions as a cell adhesion, cell migration and pathogen-binding protein [41]. The extracellular domain of CD66b was replaced by anti-Muc1 scFv to target Muc1 on the cancer cells. The cytoplasmic signal peptide can be deleted, and the chimeric antigen receptor does not contain a costimulatory domain to avoid unnecessary activation and production of additional cytokines. The mechanism by which M-DCs^TNF^ in treating cancers is also different from CAR-macrophages, of which the antitumor therapeutic effect depends on their expression of pro-inflammatory cytokines and chemokines, polarization of M2 to M1 macrophages and boosting T cell function [8].

M-DCs^TNF^ could target Mucin1 on normal epithelial cells in some tissues [42], but they will be enriched on cancers overexpressing Mucin1 [32–34]. M-DCs^TNF^ should not have off-target effects on normal cells even in the presence of an IAP antagonist because normal cells express low levels of IAP proteins and their proliferation and survival do not depend on IAP proteins [43, 44]. In previous experiments, we did not observe any side effects in SM-164-treated mice, including normal WT mice that were given SM-164 for 8 months.

In addition to targeting Mucin1 overexpressed in breast cancer, pancreatic cancer, B cell lymphoma, and multiple myeloma, DCs can also be engineered to express many other specific monoclonal scFvs against antigens, including alpha-fetoprotein, CA-125, Her-2, mesothelin, prostate stem cell antigen, and Claudin 18.2 on cancer cell surfaces. Furthermore, DCs can be engineered to express membrane-bound cytokines to target their receptors, such as EGFR, on cancer cells.

DCs are terminally differentiated cells and their life span can be weeks [38]. Thus, the therapeutic window for M-DCs^TNF^ along with an IAP antagonist is short, and high levels of TNF*α* should return to normal about 2–3 weeks at the end of therapy. If there were a concern with remaining M-DCs^TNF^, for example, chronic inflammation and stimulation of cancer growth due to high level of TNF*α*, a bisphosphonate could be administered because it can inhibit DC activity [45]. Bisphosphonates would also address concerns that an IAP antagonist could induce osteoporosis and secondarily increase bone metastasis by stimulating NF-κB activation and osteoclast formation [46] because they are used to treat osteoporosis and bone metastasis by inhibiting bone resorption [47, 48]. An alternative could be to give a TNF*α* blocker [49, 50] to reduce inflammation.

SM-164 is the most potent IAP antagonist to kill BC cells and degrade cIAP proteins [16]. However, other IAP antagonists, particularly those with confirmed biosafety in clinical trials [19–24], could also be used as long as their concentration in vivo can reach the threshold that kills cancer cells in combination with TNF*α*.

We noticed that M-DCs^TNF^ in combination with an IAP antagonist just slightly inhibit the growth of TNBC in the PDX model mice. The limited effect of this approach could be related to the following reasons. First, the viral particles of chimeric CD66b-Muc1 scFv and secreted TNF*α* used to infect the DCs are lowest effective amount, yielding only about 13–15% of the cells expressing the targeted molecules (Figs. 3A and 4A) probably because the crude viral supernatants were used. In future studies, we will concentrate the viruses to improve their infection efficacy, as it was reported [51]. Secondly, M-DCs^TNF^ was only injected once. We do not know if they had disappeared or lost the expression of TNF*α* sometime later. In future, we will monitor their life span in vivo and determine the optimal cell number and time interval for the repeating administration.

In summary, a one-time local injection of 2 × 10^5^ M-DCs^TNF^ can effectively treat established human breast cancer in an animal model when given with SM-164. M-DCs^TNF^ could also be used to treat pancreatic or lung cancers because they also overexpress Muc1 [35, 36]. Many other cells, including T cells and monocytes, could also be engineered to target delivery of TNF*α* to cancer cells in combination with an IAP antagonist because an IAP antagonist enables TNF*α* to rapidly induce apoptosis in a broad range of cancers [52–54].

## Conclusion

M-DCs^TNF^ can recognize breast cancer and produce TNF to induce cancer cell apoptosis rapidly when an IAP antagonist is given to degrade IAP proteins. Thus, M-DCs^TNF^ combined with an IAP antagonist can be used to treat advanced stage breast cancer and other solid cancers because an IAP antagonist enables TNF*α* to rapidly induce apoptosis in a broad range of cancers [52–54]. Particularly, M-DCs^TNF^ are not additionally activated because they do not have a costimulatory domain in the cytoplasmic tail of the CAR. Thus, M-DCs^TNF^ do not produce a wide variety of cytokines, except for the specifically overexpressed TNF, and do not cause the severe side effects like cytokine release syndrome.

## Data Availability

All data are available in the main text or the supplementary materials.

## Abbreviations

ACT: Adoptive cell therapies
BIRC: Baculoviral IAP repeat-containing protein
BLI: Bioluminescence imaging
CAR: Chimeric antigen receptor
CD66b: Cluster of differentiation 66b, carcinoembryonic antigen-related cell adhesion molecule 8 (CEACAM8)
CLD: Clodronate
DC: Dendritic cell
EGFR: Epidermal growth factor receptor
ELISA: Enzyme-linked immunosorbent assay
ER: Estrogen receptor
FACS: Fluorescence-activated cell sorting
GFP: Green fluorescent protein
GM-CSF: Granulocyte macrophage-colony stimulating factor,
Her-2: Human epidermal growth factor receptor 2
IAP: Inhibitor of apoptosis proteins
IL: Interleukin
M-DCs^TNF^: Mucin 1 directed DCs expressing TNFα
M-CSF: Macrophage-colony stimulating factor,
PBMCs: Peripheral blood mononuclear cells
PDX: Patient-derived xenograft model,
PI: Propidium iodide
SCFV: Single-chain fragment variable
TNF: Tumor necrosis factor-α
